# Increased Yield and Improved Transplantation Outcome of Mouse Islets with Bovine Serum Albumin

**DOI:** 10.1155/2012/856386

**Published:** 2012-12-09

**Authors:** Suzanne Bertera, A. N. Balamurugan, Rita Bottino, Jing He, Massimo Trucco

**Affiliations:** ^1^Division of Immunogenetics, Department of Pediatrics, John G. Rangos Research Center, Children's Hospital of Pittsburgh, University of Pittsburgh School of Medicine, Pittsburgh, PA 15201, USA; ^2^Department of Surgery, University of Minnesota, Minneapolis, MN 55455, USA

## Abstract

Isolation and transplantation of rodent islets are frequently used as a tool for predicting the behavior of new protocols for islet allotransplants in type 1 diabetes patients. Bovine serum albumin (BSA) is recognized as a protease inhibitor possibly protecting function and viability in islets. For this study, the addition of 0.2% BSA to the isolation protocol resulted in a 30% increase in islet yields while other parameters, such as viability and function, retained high islet quality. *In vivo*, a minimal mass of 70 BSA treated islets showed their ability to control glycemia levels in diabetic mice by bringing the average blood glucose to 153 ± 13.2 mg/dL compared to 288 ± 22.6 mg/dL without BSA. Our results show that the simple addition of BSA to the isolation protocol constitutes a reliable and reproducible method for increasing islet yield. Also adding BSA to the transplantation medium improves islet function *in vivo*. The method outlined here can reduce the overall number of animals needed per experiment and also reduce the time and resources needed for islet preparation.

## 1. Introduction

With the increased interest in finding a cure for type 1 diabetes, basic research is done with mouse models that require the isolation of islets of Langerhans from the pancreas. Several good methods of islet isolation have been described previously. Lacy and Kostianovsky method is most often referenced in which the pancreas is distended by injecting Hanks' balanced salt solution (HBSS) into the bile duct [1]. The pancreas is removed from the mouse, cut into small pieces and digested with collagenase. They further described the use of a sucrose density gradient to facilitate purification of islets from acinar tissue. Lindall et al. proposed the use of Ficoll instead of sucrose as a separation agent with the assumption that Ficoll provided a more physiologically osmotic environment for the islets [2]. 

Gotoh et al. [3] improved this method by injecting the collagenase directly into the pancreas through the bile duct then separating the endocrine from the exocrine tissue with a discontinuous Ficoll gradient. With these techniques, isolation of mouse islets has become a standard procedure used in many research laboratories. However, some mouse strains lend themselves to islet isolation less well than others. Also, some genetically altered lines of mice do not breed abundantly and each mouse is therefore precious. Using mice such as these for islet studies could benefit from a way to increase the islet yield of each mouse. Additionally, reduction, refinement, and replacement (the 3Rs) of animal use in scientific studies are strongly recommended by OLAW guidelines and IACUC committees when planning the protocol for any experiment. Although the use of mammalian pancreatic islets in diabetes research cannot be replaced by nonbiological or computer generated models at the present time, the modifications outlined here constitute a refinement of the standard islet isolation technique resulting in a reduction in the number of mice needed to obtain the same amount of islets. By adding BSA to the tissue washing medium, we routinely obtain a greater number of islets per mouse than was possible with the original protocols. A reduction in the number of islets needed for transplantation is also possible by adding BSA to the transfer solution in two ways. First, fewer islets are lost in delivery to the transplantation site due to adherence to the transfer tubing, and second, BSA protects the islets from protease damage during and immediately after graft placement. 

## 2. Materials and Methods

### 2.1. Experimental Animals

Several mouse strains were utilized in our experiments. They include 6 female nonobese diabetic mice (NOD) (8 weeks old) and 8 female NOD mice (9 weeks old) (before diabetes onset); 4 C57Bl/6 male mice, 10 weeks old; 4 C57Bl/10 female mice, 10 weeks old; 6 C57Bl/10 females at 18 weeks of age; 4 B6/129 hybrid female mice at 19 weeks of age; 6 FVB females at 20 weeks of age; 4 BALB/c males, 8 weeks old. Mice were obtained from Jackson Laboratories (Bar Harbor, MA, USA), or bred in the AAALAC (Association for Assessment and Accreditation of Laboratory Animal Care) certified Rangos Research Center Animal Facility. The donor and recipient mice in the transplant study were ICR females, 8–10 weeks old obtained from Taconic Farms (Germantown, NY). All mice are housed in microisolator caging within National Institute of Health guidelines for animal care in a specific pathogen free environment. The University of Pittsburgh IACUC approved the animal protocol used in this study.

### 2.2. Isolation and Transplantation Reagents

Tissue culturing media consists of RPMI 1640 media supplemented with 20 mM Hepes, 1% L-glutamine, 1% penicillin/streptomycin, 50 *μ*M b-mercaptoethanol (2-ME), and 10% heat-inactivated fetal bovine serum. Whole islets were maintained in complete medium with a density of ~150 islets in 5 mL medium at 37°C in an atmosphere of 95% air and 5% CO_2_. Media was replaced every 2-3 days. Tissue culture reagents used in this study were purchased from BioWhittaker (Walkersville, MD, USA) and Life Technologies (Grand Island, NY, USA). Collagenase Type V, 2-ME, streptozotocin, and reagents for Kreb's-Ringers Buffer (KRB) were purchased from Sigma (St. Louis, MO, USA). Due to variability in the activity of different lots of collagenase, all collagenase used in this study was from a single lot. KRB consisted of 115 mM NaCl, 24 mM NaHCO_3_, 5 mM KCl, 2.5 mM CaCl_2_, 1 mM MgCl_2_, 25 mM Hepes, 0.1% BSA, and either 2.8 mM or 20 mM glucose. Hanks' Balanced Salt Solution with calcium and magnesium was supplemented with either 20 mM Hepes (HBSS-Hepes) or 20 mM Hepes plus 0.2% BSA (HBSS-BSA) then filter sterilized. 

### 2.3. Ficoll Preparation

Ficoll was made from Ficoll Type 400 (Sigma, St. Louis, MO, USA) and was dissolved at the rate of 33.35 g Ficoll per 100 mL HBSS-Hepes. This concentration produces a 25% stock solution that is filter sterilized and stored at 4°C. Gradient densities are made in 50 mL aliquots from the 25% Ficoll stock as follows: 23% (4 mL HBSS-Hepes plus 46 mL stock), 20.5% (9 mL HBSS-Hepes plus 41 mL stock), and 11% (28 mL HBSS-Hepes plus 22 mL stock). All gradients are mixed thoroughly and stored at 4°C.

### 2.4. Organ Harvest

**Figure 1:**
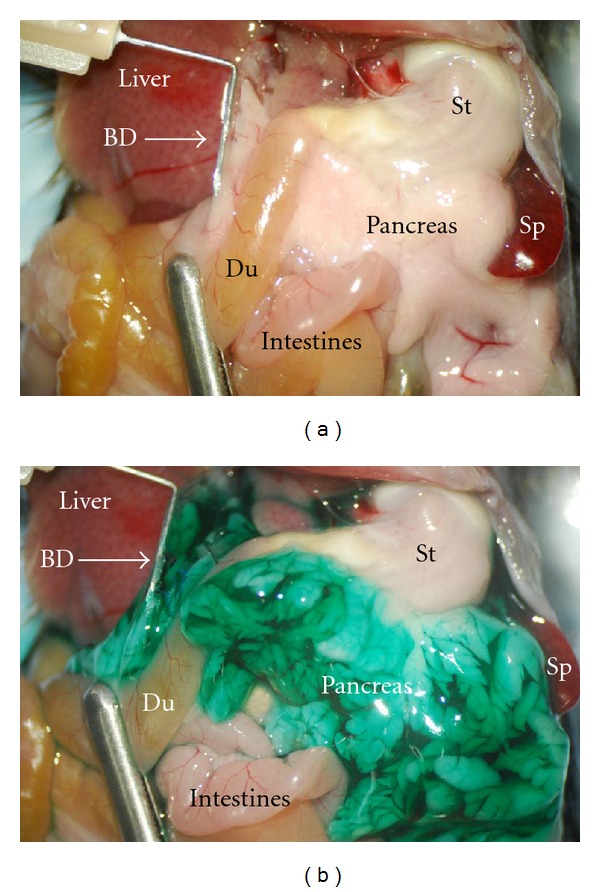
Distention of pancreas with collagenase solution. (a) Before injection of collagenase, a bulldog clamp (silver bar at bottom left) is placed around the duodenum (Du) and over the Papilla of Vater of the common bile duct to prevent drainage into the duodenum. The common bile duct (BD) is cannulated with angled hypodermic needle (upper left of picture) in preparation to suffuse pancreas with collagenase. The pancreas is the whitish mass between the spleen (Sp), stomach (St), liver, and intestines. (b) Same picture after inflation. A green vegetable dye is used here to visualize full distension of the pancreas.

Islets of Langerhans are separated from the pancreas by digestion of the tissue with collagenase as previously described [4, 5]. The procedure was carried out under sterile conditions as follows. The animal is sacrificed immediately before harvest. A laparotomy is performed, and the liver reflected back against the diaphragm. The common bile duct is located and clamped at the papilla of Vater (Figure 1(a)). A 27G or 30G needle (depending on the size of the duct) is inserted into the bile duct (Figure 1(a)), and 2-3 mL cold collagenase solution (1.95 mg/mL in HBSS-Hepes) is injected until the pancreas is visibly distended (Figure 1(b)). 

 The pancreas is then removed by gently tearing/cutting away from the attachment points. For maximum yield, attention should be given to removing the entire pancreas. Additionally, since the presence of blood reduces enzyme activity, the excised organ is washed briefly in cold collagenase solution to remove any residual blood. Blood clots are also removed. The organ is then placed into a sterile 25 cm^2^ tissue culture flask (Corning Inc., Corning, NY, USA) and kept on ice. Up to five pancreata may be placed in one flask. An additional 2-3 mL collagenase solution is added to each flask. For our comparisons, only pancreata that inflated completely were used. Warm ischemia is held to a 5 minute maximum.

### 2.5. Organ Digestion and Tissue Washing

The excised, collagenase infused pancreata are incubated for 18–20 minutes at 37°C without shaking. Immediately after removing the flask from the incubator, the flask is shaken sharply for 5–10 seconds to homogenize the tissue. The digestion is stopped by diluting the homogenized pancreatic tissue with 10 mL cold HBSS-Hepes. For our comparison, the digested tissue was equally divided at this point and poured into separate 50 mL conical tubes and washed 3–5 times to remove small, light-weight tissue particles. The washes are performed by adding either cold HBSS-Hepes (control group) or HBSS-BSA (experimental group) to the digested tissue up to 50 mL after which the tissue particles are allowed to gravity settle for 5 minutes before removing the supernatant. Centrifugation between washes is not done. Approximately 45 mL of the wash supernatant is carefully aspirated and discarded without disturbing the loose pellet. The pellet contains the heavier fragments, including the islets. After resuspending the pellet in the remaining solution, the tube is filled again and the washing steps are repeated until the supernatant is relatively clear of the fine tissue fragments. 

### 2.6. Discontinuous Ficoll Gradient

After the supernatant is removed from last wash, the remaining tissue is resuspended and placed into sterile round bottom 17 × 100 mm polystyrene culture tubes with caps (Fisher, Pittsburgh, PA, USA), so that there was the equivalent of no more than 2 organs per tube and centrifuged gently for 1 minute at 1000 RPM. After pelleting the tissue, as much as possible of the supernatant is removed so as not to dilute the 25% Ficoll solution. The discontinuous gradient is constructed by first resuspending (by vortex) the pelleted pancreatic tissue in 4 mL of the 25% Ficoll stock solution. Subsequently, two mL each of the other gradients (23%, 20.5%, and lastly 11%) are layered in order of highest density to lowest, over the 25% Ficoll/tissue mixture. Each tube therefore contains a total of 10 mL with the pancreatic tissue suspended in the 25% fraction at the bottom. The gradient preparation is centrifuged for 10 minutes at 1800 RPM. The majority of islets are usually found at the 11%–20.5% and/or the 20.5%–23% interfaces. The layers are then separated and put into 15 mL conical tubes, washed with HBSS-Hepes or HBSS-BSA, and centrifuged for one minute at 1000 RPM. The supernatant is aspirated, and the islets washed again and allowed to gravity settle to remove the last traces of Ficoll. All but 1 mL of the supernatant is removed, and the remaining 1 mL, containing the islets, is transferred to a sterile, nontissue culture-coated 60 mm Petri dish (Fisher Scientific, Pittsburgh, PA, USA) containing 5-6 mL of complete medium. Most of the islets are found in the top 2-layer interfaces but some may also be found in the lower 25% layer. Therefore, all layers are washed and examined for islets before discarding. The islets are then handpicked to eliminate any remaining exocrine tissue and counted. Whole islets are maintained in complete media at 37°C in a humidified atmosphere of 95% air and 5% CO_2_.

### 2.7. Size and Enumeration of Recovered Islets

**Figure 2:**
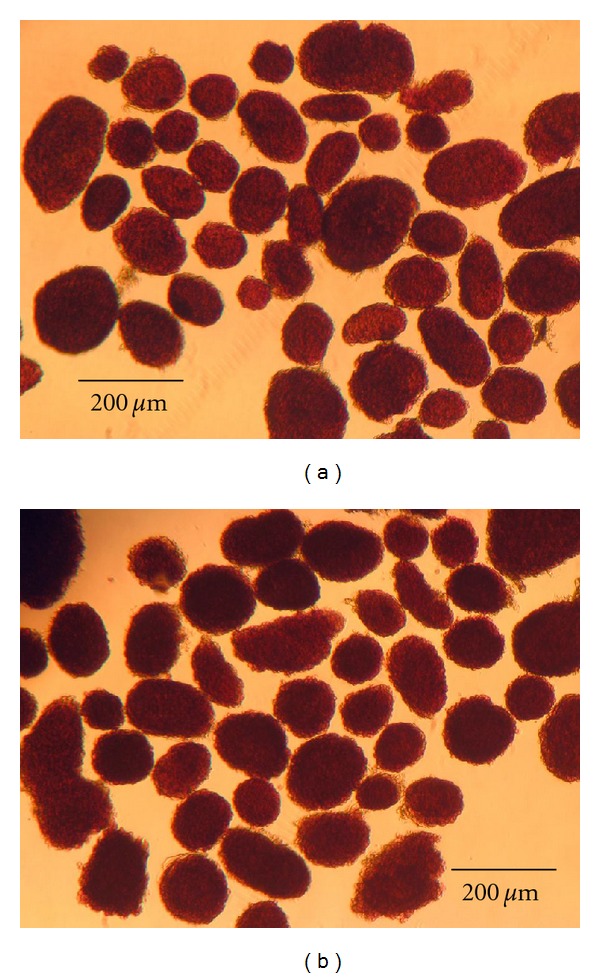
Normal mouse pancreatic islets after collagenase digestion and Ficoll separation with further purification by handpicking. Islets were stained with dithizone (red). The left panel shows islets isolated with BSA; the right panel shows islets isolated without BSA. Close to 100% purity may be achieved with this method. Scale bar indicates size in microns.

Individual mouse islets may range from smaller than 50 to over 400 microns in size **(** Figure 2). For the purpose of this study, islets larger than 50 microns were counted by direct enumeration. Islets smaller than 50 microns were counted by combining 2–4 islets depending on size, and counting them as 1 islet. The number and size of islets recovered varies with strain and age of the animal with retired female breeders having the largest and most per animal. The adult animals used in this study generally had islets in the same proportionate size ranges. In general, the size range of the individual islets are approximately 20% less than 50 *μ*m, 30% between 50 and 100 *μ*m, 35% between 100 and 250 *μ*m, and 15% greater than 250 *μ*m. On average, this isolation method reliably recovers 150–300 islets from an adult mouse (25 g body weight or larger). Islets were counted immediately after isolation. For transplantation experiments, islets of size 100–200 *μ*m were used to assure uniform islet mass between recipients.

### 2.8. Islet Functional Assessment-Perifusion Assay

Insulin release in response to changing glucose conditions was determined with a perifusion assay for at least three different islet preparations [6]. For the assay, 75 islets of similar size from each of the BSA and non-BSA-treated islet groups were handpicked and cultured identically overnight in complete medium at 37°C and 5% CO_2_. After a 30 minute preincubation period, the islets were perifused for 30 minutes with KRB containing a subphysiological (low) glucose concentration (2.8 mmol/l) followed by a 30 minute exposure to high-glucose conditions (20 mmol/l) and returned to subphysiologic conditions for a 30 minute recovery period. Elution samples were taken once per minute for 90 minutes. Insulin concentration of the elution samples was determined by mouse insulin ELISA (Alpco, Windham, NH, USA).

### 2.9. Viability Assay

Calcein-AM (1 *μ*L) (Molecular Probes, Eugene, OR) and propidium iodide (PI, 10 *μ*L) (Sigma-Aldrich, St Louis, MO, USA)are added to 1 mL PBS and protected from light. Islets are added to the mixture and incubated at 37°C for 25 minutes. Islets are examined by fluorescent microscopy immediately following incubation. Image analysis of the percentage of dead cells (PI stained red) to live cells (Calcien-AM stained green) is calculated using Metaphor Image Analysis software (Molecular Devices, Downingtown PA, USA). The area of PI positive stain to the total area of the islets is measured. Results are expressed as the percentage of dead cells in the islet group.

### 2.10. Antibody Staining of Islets

Islets are cytospun onto gelatin-coated slides. After a 5-minute fixing step in 2% paraformaldehyde in PBS, slides are blocked with 20% nonimmune normal donkey serum for 1 hour at room temperature. Primary polyclonal rabbit anti-insulin (1 : 100) and goat antiglucagon (1 : 50) antibodies (Santa Cruz Biotechnology, Inc. Santa Cruz, CA, USA) are double immune stained on these samples. Secondary antibodies Alexa Fluor 488 donkey anti-rabbit (Molecular Probes, Eugene, OR, USA) and Cy3-conjugated donkey antigoat (Jackson Immuno Research Lab, INC. West Grove, PA, USA) are used. For duct cell staining, Dolichos biflorus agglutinin lectin (FITC-DBA) (1 : 100) (Vector Lab, Inc. Burlingame, CA, USA) is applied then the islets are treated with Cy3-conjugated donkey anti-insulin secondary antibody. Slides are washed 3 times and mounted with cover glass. Images are viewed at 400x magnification and captured with a Nikon confocal microscope (Nikon D-ECLIPSE C1, Japan). 

### 2.11. Insulin Extraction

Prior to perifusion, aliquots of 10 islets for each experimental and control group are ultrasonically disrupted in 0.2 mL double distilled water. A 50 *μ*L fraction of the homogenate is mixed with 125 *μ*L acid ethanol (0.18 M HCl in 96% (vol/vol) ethanol), and the insulin is extracted overnight at 4°C. Insulin is measured by ELISA (Alpco, Windham, NH, USA).

### 2.12. Streptozotocin Administration

Prior to islet transplantation, the recipient animals are rendered diabetic with a single high dose (200–250 mg/kg) of Streptozotocin (STZ) (Sigma-Aldrich, St. Louis, MO, USA) diluted in saline with thorough mixing [4]. Saline is preferred over the citrate buffer because saline causes less distress to the mouse than injecting an acidic solution. Dissolved STZ is kept at 4°C for 1-2 hours then mixed again before IP injection. Animals become diabetic at a similar rate with saline as with citrate buffer. Diabetes is confirmed by sustained blood glucose levels above 350 mg/dL for 2 or more consecutive days. All transplant recipients used in this study have pretransplant nonfasting blood glucose levels between 350 and 450 mg/dL. Diabetic animals are maintained with insulin injections and Ringer's lactate solution.

### 2.13. Islet Transplantation

Animals are transplanted as previously described [4, 5]. Briefly, the animals are anesthetized with 2.5% Avertin, and a small incision is made in the flank about one-half inch to the left of midline. The left kidney is located and externalized. A small hole is made at one pole of the kidney capsule by gently tearing it with fine tipped no. 5 watchmaker's forceps (Roboz, Rockville, MD, USA). The islets were washed in either HBSS-BSA or HBSS-Hepes and aspirated into a 3 to 4 inch piece of PE no. 50 equivalent tubing (Harvard Apparatus, Holliston, MA, USA) then gently centrifuged to a loose pellet. The tubing is inserted into the space between the capsule and the kidney, and the islets are slowly expelled as the tubing is withdrawn (see Figure 6). The kidney is replaced in the abdominal cavity, and the body wall is sutured with 6-0 nylon before the outer skin is closed with small wound clips. The animals are kept warm until recovery from anesthesia. 

### 2.14. Statistical Analysis

Student's two-tailed *t* test for paired samples is applied to the islet data to determine significant statistical probability. By conventional criteria, *P* values of <0.05 are considered significant.

## 3. Results

### 3.1. Number of Islets Obtained

The digested pancreatic tissue was separated into 2 equal portions, and the islets were separated either with or without BSA in the washing medium.  Table 1 shows the number of islets obtained from these 2 groups, as well as the strain, age, sex, and number of mice used per group. The NOD female mice were not yet diabetic, and had blood glucose readings were between 57 and 145. Islets were counted by 3 observers blinded to the identity of the groups. In every comparison, more islets were obtained from the BSA treated groups than from those processed without BSA. The total number of islets obtained in the BSA group was 4577 ± 119 (*n* = 21) while the number recovered without BSA was 3207 ± 97 (*n* = 21). Overall, the difference between the islet totals were statistically significant with a *P*-value of 0.004. 

### 3.2. Assessment of Islet Function and Insulin Content

**Figure 3:**
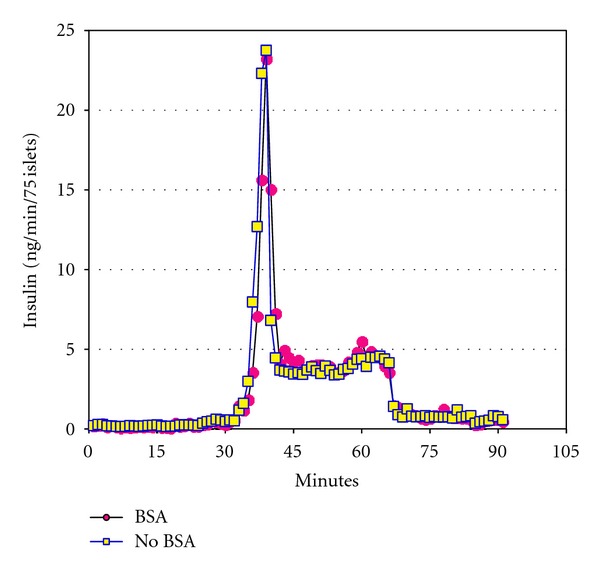
Perifusion assay after 24 hours in culture. A dynamic perifusion assay for insulin response was performed with 75 islets in each group. After a 30 minute preincubation step, islets were exposed for 30 minutes to low glucose (2.8 mM) then for 30 minutes to high glucose (20 mM) and back to 30 minutes of low glucose. Circle shapes represent the BSA group and square shapes represent the group with No BSA. Graph shown was one of 9 assays performed. There was no significant difference between the 2 groups for insulin response to glucose at 24 hours.

Dynamic insulin secretion from low (2.8 mM) and high (20 mM) concentrations of glucose is measured 24 hours after isolation either with or without BSA and showed no significant difference in the way the islets responded to varying concentrations of glucose. One of these results is shown in Figure 3. Insulin content of the islets was determined from 9 different isolations. For the BSA group, the mean and SD were 78.06 ng ± 45.62 while the No BSA group had 87.41 ng ± 46.62. These differences were not statistically significant.

### 3.3. Viability Assay

**Figure 4:**
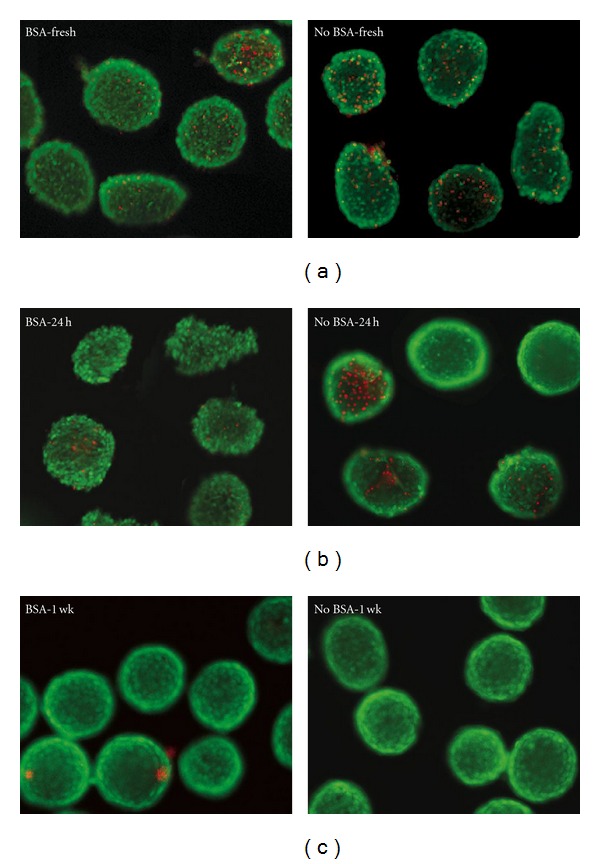
Viability assay using Calcien-AM (green) to indicate live cells and Propidium iodide (red) that stains dead cells. Top panel shows islets stained 1 hour after isolation, middle panel is after 24 hours in culture, and bottom panel is after 1 week in culture. Original magnification 200x.

The islets were stained for viability at 1 hour, 24 hours, and 1 week after isolation. No significant differences were seen between the 2 groups at any time point. Typically, with our culture conditions, 7–10% of the larger islets are lost after overnight culture in both groups and commonly have centrally located dead cells (data not shown). For the fresh islets, the BSA group had an average of 4.9% dead cells compared to 5.7% in the No BSA group. At the 24 hr time point, the islets isolated without BSA had a slightly higher percentage of dead cells (6.0%) than the BSA group (2.0%) (Figure 4). After one week, the dead cells in the BSA group were 2.7% while those in the No BSA group were 4.5%. None of these differences were statistically significant with *P*-values of 0.744 (Fresh), 0.146 (24 h), and 0.442 (1 week). 

### 3.4. Insulin, Glucagon, and Duct Cell Staining

**Figure 5:**
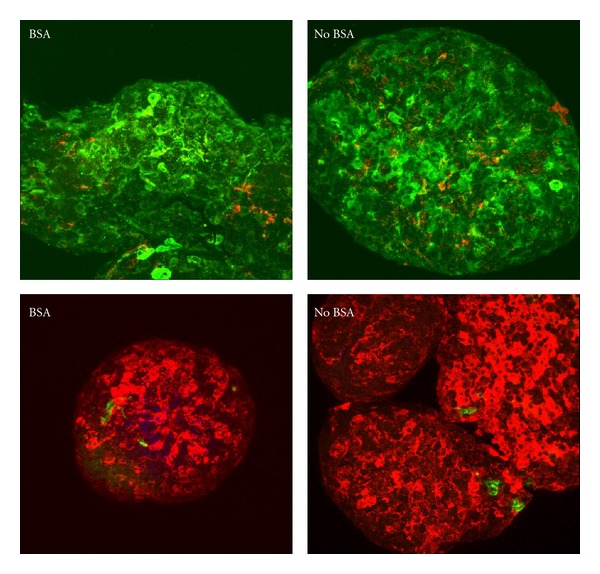
Left hand panels show islets isolated with BSA while right hand panels show islets isolated without BSA. The islets were fixed in 4% PFA and cyto-spun onto glass slides before staining. Islets in top panels were stained for insulin (green) and glucagon (red). Bottom panels instead show insulin stained in red while duct cells were stained in green. Original magnification 400x.

The top panels in Figure 5 show islets from each of the BSA or No BSA groups that were stained with antibodies for insulin (green) and glucagon (red). Insulin staining was abundant in both groups, and the amount of glucagon positive (alpha) cells were comparable with approximately 15–20% in either group. The lower panels in Figure 5 show insulin in red this time with DBA positive (duct cells) in green. A small number of duct cells per islet were detected and found to be consistent between the groups.

### 3.5. Islet Transplantation Results

**Figure 6:**
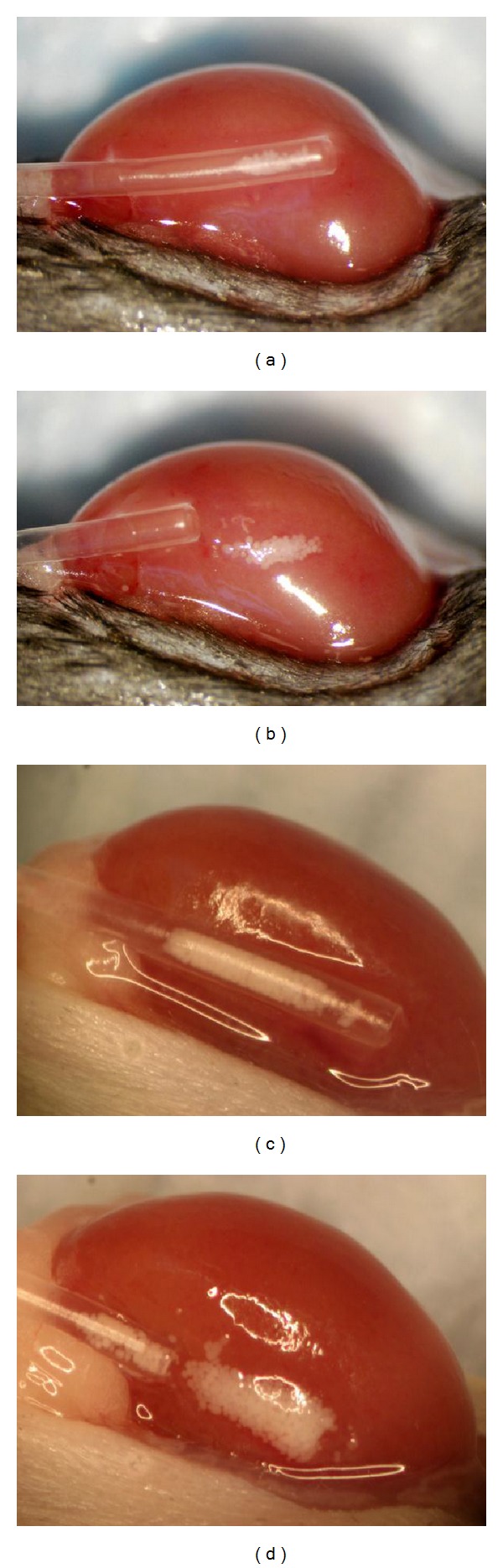
Islet transplantation under the kidney capsule. (a) Mouse kidney with PE50 tubing containing 70 syngeneic islets in HBSS-BSA solution. (b) Islets are expelled from the tubing and deposited under the capsule with no islets remaining adherent inside the tubing. (c) For comparison, islet transplant using 400 islets frequently required for grafts transplanted without BSA to achieve normalization. (d) Some islets adhere to the tubing after depositing the majority under the kidney capsule. Original magnification 65x.

Streptozotocin-induced diabetic mice were transplanted with 70 syngeneic islets under the left kidney capsule.  Figure 6(a) shows the islet mass in BSA solution in the tubing inserted under the kidney capsule.  Figure 6(b) shows the islets after being deposited under the capsule. This minimal mass of 70 islets was visually compared to a graft consisting of 400 islets, (Figures 6(c) and 6(d)) a number typically used to achieve euglycemia. The 400 islets were trnsplanted in a solution that did not contain BSA. Note that some of the islets adhered to the tubing (Figure 6(d)) and did not get deposited under the capsule.

**Figure 7:**
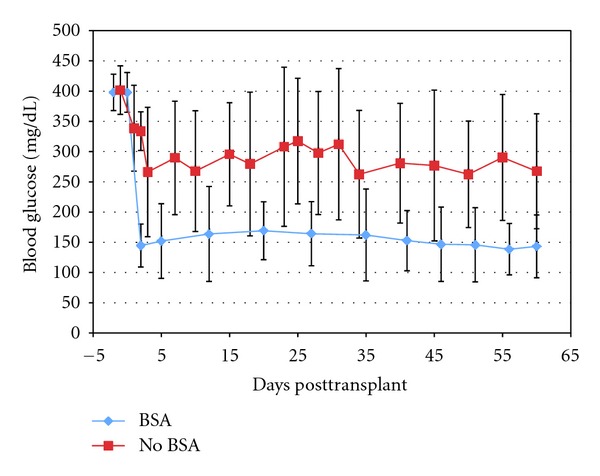
Glycemic levels in transplanted mice. Glycemic levels (mg/dL) of Streptozotocin treated mice were followed for 2 months after syngeneic transplant of 70 islets. Without BSA (*n* = 11, squares); with BSA (*n* = 18, diamonds). Transplant was performed on day 0. The difference in average glycemia between the groups was statistically significant (*P*-value 7.8^−11^).

A total of 29 diabetic mice with nonfasting glycemia between 350 and 450 mg/dL were used as transplant recipients. Of these, 18 mice were transplanted with islets isolated with BSA and had BSA in the transplantation solution. The other 11 mice were transplanted with islets isolated without BSA and without BSA in the transplantation solution. To ensure the entire graft was transplanted, the transfer tubing was inspected for any remaining islets. Only recipients receiving the full amount of islets under the kidney capsule were included in the study. The mass of each graft in both groups was also examined to confirm an equivalent islet mass for every transplant. In the BSA group, 17/18 or 94% reached an average non-fasting blood glucose level of approximately 150 mg/dL by day 2 compared to 0/11 mice without BSA. Glycemia levels were monitored for 60 days at which time 16/18 or 89% of the mice in the BSA group had a sustained non-fasting blood glucose level near 150 mg/dL while none (0/11) in the No BSA group reached that level (Figure 7). The average blood glucose level of the BSA group was 153 ± 13.2 mg/dL compared to the average for the no BSA group of 288 ± 22.6 mg/dL. These results are statistically significant with a *P*-value of 7.8^−11^.

## 4. Discussion

The improvement of our method to the classically cited isolation protocols is immediately evident as a 30% increase in islet yield. Our method of manual islet isolation is based on the technique outlined by Gotoh et al. [7] with the addition of BSA to the washing solutions. Citing Carre and Lacarriere, the increased yield resulting from this simple addition is most likely due in part to the adsorption of BSA protein by the cells that results in the islets unable to adhere to plastic surfaces. These authors also demonstrated that using medium supplemented 10% fetal bovine serum (FBS) (FBS contains 15 g/l BSA) resulted in 45% of CHO cells adhering to the plastic, while no cells adhere with BSA alone, and postulate that the other components in the FBS overcome some of the antiadhesion effects of the BSA [8]. Therefore, for our purpose, BSA is preferred over FBS to use in the islet isolation process.

The other parameters we studied, that is, viability, composition, and response to glucose, showed no significant differences between the islet groups. However, one reason for the lack of difference in islets after they have been cultured may be due to the FBS in the culture medium acting as a protein source and as a protease inhibitor to protect the islets from residual digestive enzymes [9]. 

The transplantation data clearly shows an advantage for islets that have been isolated and transplanted with BSA, over islets that did not have a serum source during these procedures. Although control islets were cultured in serum-containing media for a short period (24–48 hours) before transplantation, they could not recover sufficiently in that time to be fully functional in a continuous, high-demand situation such as that occurs after transplantation into a diabetic animal. Also, the ability of BSA to prevent cell adherence proves beneficial during graft transfer. As shown in Figure 6(b), every islet to be transferred was placed under the capsule, and none were left in the tube because of attachment to the plastic. To the contrary, there were some islets that remained in the tubing after transplantation without BSA (Figure 6(d)).

Studies with rat islets indicated that BSA possibly acts as a protease inhibitor and strongly suppresses the endogenous proteolytic enzyme activity of exocrine acinar cells [10, 11]. Therefore, adding BSA to the isolation solutions also helps to protect the endocrine islets from the deleterious effects of proteases released after collagenase digestion. Because of this protection, the tissue can be washed extensively, not only to remove remaining traces of collagenase, but also to remove small tissue fragments to improve islet purity after Ficoll separation. 

BSA has also been proposed as a separation gradient. In a short article from 1986 and followup in 1987, Lake and associates compared a BSA gradient to a Ficoll gradient. Testing with rats, it was found that more islets were obtained with BSA than Ficoll (714 to 368 islets per rat pancreas) and that there was less exocrine tissue contamination with BSA than with Ficoll prepared islets [12, 13]. A study with monkeys also obtained more islets with BSA as a separation gradient than Ficoll (862 ± 96 to 752 ± 101) but found that dextran had the best yield of the three (1038 ± 81) [14]. Field et al. also reported similar results again in rats, by adding 35% BSA to a Dextran separation gradient [15]. Their yield was 1222 islets per rat pancreas to 850 with dextran alone. More recently, Zmuda et al. published an isolation protocol using Histopaque for separation [16], and McCall et al. [17] did a comparison of separation media that concluded Histopaque was as good as Ficoll. In light of these findings, we attempted to improve the separation media by adding BSA to the Ficoll but found no advantage. In our hands, when BSA was added to the Ficoll, there was a greater amount of acinar tissue contamination in the final preparations than with Ficoll alone (data not shown). We also tried adding BSA to the collagenase solution as suggested in several studies for rat islets [18, 19], but the overall outcome in our mouse study was not improved (data not shown). 

## 5. Conclusions

 We maintain that the addition of BSA to the washes during isolation was alone sufficient to increase the number of mouse islets recovered. We were, therefore, able to reduce the number of mice needed for islet isolation and transplantation with the refinements described herein. The total number of islets we obtained from various strains of mice using HBSS-BSA during the isolation procedure was 4577 ± 49.1 compared to 3207 ± 18.9 for those isolated without BSA (*n* = 21 mice/group). This constitutes a statistically significant (30%) increase in islet yield when 0.2% BSA was added to the wash medium. For transplants, the number of islets needed for reliable normalization can be significantly reduced from the average of 400 that is commonly used, and consequently, the number of donors required for islet transplantation can also be reduced. 

## Figures and Tables

**Table 1:** Number of islets obtained with and without BSA.

				Number of Islets/treatment group	
Strain	Age (wks)	Sex	*n*/group	With BSA SD	Without BSA SD
C57Bl/6	10	M	2	450 ± 18.2	335 ± 20.0
B6/129	19	F	2	516 ± 27.4	382 ± 11.3
BALB/c	8	M	2	436 ± 27.2	304 ± 13.6
NOD	8	F	3	625 ± 17.6	245 ± 6.0
NOD	9	F	4	685 ± 15.5	540 ± 12.1
C57Bl/10	10	F	2	330 ± 10.6	275 ± 11.9
C57Bl/10	18	F	3	595 ± 30.5	436 ± 12.5
FVB	20	F	3	940 ± 25.2	690 ± 19.7

		Totals	21	4577 ± 119*	3207 ± 97*

Islets were isolated by first distending the pancreas with collagenase solution (1.95 mg/mL Type V). The tissue was then digested and separated into 2 equal portions. One portion was processed with BSA in the washes throughout the isolation process and the other without BSA in the washes. Several strains of mice were used and their age in weeks, sex and number of mice in each treatment group (*n*/group) are indicated.

*P = 0.004.
